# Aqueous humor exosomal microRNAs as emerging biomarkers in HSV-1 anterior uveitis

**DOI:** 10.3389/fcimb.2026.1986644

**Published:** 2026-10-21

**Authors:** Maria Carmela Saturno, Dimitrios Kalogeropoulos, Elena Carbone, Paola Piscopo, Oscar Matteo Gagliardi, Maurizio La Cava, Alessandro Lambiase, Danilo Iannetta

**Affiliations:** 1 Department of Sense Organs, Sapienza University of Rome, Rome, Italy; 2 Bristol Eye Hospital, University Hospitals Bristol and Weston NHS Foundation Trust, Bristol, United Kingdom; 3 Department of Neuroscience, Istituto Superiore di Sanità, Rome, Italy

**Keywords:** anterior uveitis, aqueous humor, biomarkers, exosomes, extracellular vesicles, HSV-1, microRNAs, ocular immunovirology

## Abstract

Herpes simplex virus type 1 (HSV-1) anterior uveitis is a recurrent inflammatory disease in which aqueous humor (AH) polymerase chain reaction (PCR) can provide virological confirmation but offers limited information on the molecular features of the intraocular environment. Extracellular vesicles (EVs) are constitutively present in the physiologically acellular AH and carry selectively packaged molecular cargo, including microRNAs (miRNAs), making them a relevant source of local biological information. This narrative review examines the rationale for investigating AH-derived EV-associated miRNAs in HSV-1 anterior uveitis by integrating evidence from ocular EV research, miRNA studies in uveitis and experimental models of HSV-1 infection. Studies in ocular disease have shown that AH-derived EVs can be isolated and molecularly characterized despite the small sample volumes available, while systemic and intraocular miRNA profiling has identified disease-associated signatures across several forms of uveitis. Experimental HSV-1 studies further demonstrate that both host- and virus-derived miRNAs regulate viral replication, latency and ocular immunopathology. In infectious uveitis, comprehensive AH miRNA profiling has already been shown to be technically feasible. To date, no study has directly characterized AH-derived EV-associated miRNAs in HSV-1 anterior uveitis. Their investigation is therefore supported by biological plausibility and indirect methodological evidence and may complement viral DNA detection by providing additional information on the local intraocular molecular environment.

## Introduction

Herpes simplex virus type 1 (HSV-1) anterior uveitis is one of the leading causes of anterior uveitis, characterized by a chronic relapsing course driven by recurrent episodes of viral reactivation. Repeated intraocular inflammation may progressively lead to corneal endothelial dysfunction, iris atrophy, ocular hypertension, secondary glaucoma and subsequent irreversible visual impairment (Babu et al., 2020; Standardization of Uveitis Nomenclature (SUN) Working Group, 2021; Han et al., 2026).

The diagnosis of HSV-1 anterior uveitis is mostly based on the clinical phenotype, while aqueous humor (AH) polymerase chain reaction (PCR) can offer virological confirmation in selected cases. PCR enables detection of HSV-1 DNA and can be useful when the clinical features overlap with other infectious or non-infectious etiologies of anterior uveitis. PCR for HSV has a reported sensitivity of 91.3% and a specificity of 98.8% (Standardization of Uveitis Nomenclature (SUN) Working Group, 2021). To maximize its diagnostic yield, AH sampling should be performed during active inflammation, when the presence of inflammatory cells in the AH may increase the possibility of detecting intracellular viral DNA. Nevertheless, a negative result does not exclude a viral etiology because PCR sensitivity may be influenced by low intraocular viral load, limited sample volume, the timing of aqueous sampling, rapid clearance of viral DNA and the presence of inhibitory compounds within the specimen (Babu et al., 2020; Méndez-Rodríguez et al., 2025). Furthermore, although AH PCR provides substantial virological information, it provides little information regarding local immune activation, disease activity or the molecular mechanisms underlying recurrence (Babu et al., 2020; Standardization of Uveitis Nomenclature (SUN) Working Group, 2021; Méndez-Rodríguez et al., 2025; Han et al., 2026).

Extracellular vesicles (EVs), including small EVs frequently referred to as exosomes, have emerged as significant mediators of intercellular communication and promising sources of biomarker information. By transporting several molecules, EVs reflect the physiological and pathological state of their cells of origin, participating in immune regulation, inflammation and host–pathogen interactions (Dismuke et al., 2015; Aziziyan et al., 2026; Liao et al., 2026).

Among EV cargo molecules, microRNAs (miRNAs) are of particular interest, modulating post-transcriptional gene expression. During HSV-1 infection, miRNA networks orchestrate key events of the viral life cycle, including latency, viral replication, immune evasion and inflammatory responses (Umbach et al., 2008; Pan et al., 2019; Duan et al., 2023).

To date, most studies investigating miRNAs in ocular inflammatory diseases have focused on peripheral blood, plasma-derived EVs or peripheral blood mononuclear cells, providing valuable insights into systemic immune dysregulation but only indirect information about intraocular molecular events (Yang et al., 2017; Duraikkannu et al., 2023; Jiang et al., 2023; Sharon et al., 2025). By contrast, AH is in direct contact with inflamed anterior segment tissues and therefore represents a particularly relevant biofluid for investigating local molecular responses. Recent advances in EV isolation and ultra-low-input small RNA sequencing have made comprehensive molecular profiling feasible even from the small aqueous humor volumes routinely obtained during diagnostic paracentesis (Chadalawada et al., 2022; Aziziyan et al., 2026).

However, the current literature on AH-derived EVs remains methodologically heterogeneous. Available studies differ substantially in sample volume, EV isolation and enrichment strategies, vesicle characterization, RNA extraction protocols, sequencing depth and downstream analytical pipelines. In addition, small AH volumes increase the risk of low EV and RNA yield, while commonly used isolation methods may co-isolate non-vesicular extracellular components, potentially affecting molecular readouts (Mateescu et al., 2017; Van Deun et al., 2017; Théry et al., 2018; Jeppesen et al., 2019). Sample heterogeneity and the limited size of most published cohorts further restrict direct comparison across studies. Therefore, existing ocular studies should be regarded as methodological proof of principle rather than definitive evidence that AH EV-associated miRNA profiling is analytically standardized or clinically validated.

The anterior chamber represents a highly compartmentalized and immunoregulatory microenvironment in which resident ocular cells, infiltrating leukocytes and virus-infected tissues may contribute differently to the extracellular vesicle pool. Ocular barriers may limit the equilibration of locally released EVs and miRNAs with the systemic circulation, potentially enriching AH for molecular signals generated within the eye. At the same time, local immune regulation and inflammatory activity may influence EV secretion, cargo composition, trafficking and clearance (Liu et al., 2020; Rudraprasad et al., 2022; Aziziyan et al., 2026; Han et al., 2026). Consequently, AH-derived EV-associated miRNAs may provide information more closely related to local intraocular events than peripheral miRNA profiles, although the extent to which they reflect specific cell populations or disease processes remains to be established.

To our knowledge, no study has yet characterized the EV-associated miRNA profile of AH in patients with HSV-1 anterior uveitis. The rationale for investigating this approach therefore derives from converging evidence from related ocular diseases, infectious uveitis and experimental HSV-1 models rather than from direct evidence in HSV-1 anterior uveitis. This narrative review critically evaluates this biological and methodological rationale, discusses current limitations and knowledge gaps, and outlines priorities for future translational research.

## Methods

A structured literature search was conducted to identify studies investigating extracellular vesicles, exosomes, miRNAs and their role in ocular inflammation and HSV-1 infection. A literature search was performed in PubMed, Scopus and Web of Science from database inception to 31 July 2026 using combinations of keywords related to extracellular vesicles, exosomes, microRNAs, aqueous humor, ocular biofluids, uveitis, ocular inflammation, HSV-1 and anterior uveitis. Additional relevant publications were identified through manual screening of the reference lists of eligible articles and pertinent review papers.

Considering the absence of direct studies on AH-derived EV-associated miRNAs in HSV-1 anterior uveitis, the search strategy was intentionally broad and included related ocular inflammatory and infectious disorders, as well as experimental HSV-1 studies. These data were used to establish biological plausibility, identify methodological precedents and define current knowledge gaps rather than to infer direct clinical efficacy or technical feasibility in HSV-1 anterior uveitis. Accordingly, evidence supporting the feasibility of AH EV-associated miRNA profiling in this setting should be regarded as indirect and hypothesis-generating, as no study has yet specifically evaluated this approach in patients with HSV-1 anterior uveitis.

This review considered original clinical studies, experimental and translational research and relevant mechanistic investigations addressing EV biology, miRNA regulation, ocular inflammatory diseases, HSV-1 infection and biomarker discovery. The retrieved studies were reviewed and narratively synthesized according to their relevance to the objectives of this review.

## Extracellular vesicles in ocular diseases

The diagnostic potential of EVs has been investigated across a range of ocular diseases. Unlike most biological fluids, AH is relatively acellular under physiological conditions, making EVs one of the principal sources of molecular information within the anterior chamber. Small extracellular vesicles (small EVs), frequently referred to as exosomes in the ocular literature, are released by several ocular cell types, including corneal epithelial cells, trabecular meshwork cells, retinal vascular endothelial cells and retinal pigment epithelial cells (Liu et al., 2020; Rudraprasad et al., 2022; Aziziyan et al., 2026). According to current EV nomenclature, the term “exosome” should be reserved for vesicles of demonstrated endosomal origin; therefore, “EVs” or “small EVs” is used throughout this review when vesicle biogenesis has not been directly established (Théry et al., 2018). EVs carry proteins, lipids, mRNAs and miRNAs that may reflect the physiological or pathological state of their cells of origin. Their lipid bilayer can protect enclosed nucleic acids from extracellular enzymatic degradation, potentially enhancing their stability as carriers of molecular information (Liu et al., 2020; Rudraprasad et al., 2022; Aziziyan et al., 2026). The stability of extracellular miRNAs depends strongly on the molecular compartment in which they are carried. miRNAs enclosed within EVs are relatively protected from extracellular RNase activity, whereas non-vesicular miRNAs may also remain stable through association with RNA-binding proteins such as Argonaute 2 or with lipoprotein complexes (Arroyo et al., 2011; Vickers et al., 2011). This relative resistance to enzymatic degradation supports the investigation of EV-associated miRNAs as candidate biomarkers. However, the *in vivo* half-life of individual EV-associated miRNAs in human aqueous humor has not been established and may vary according to vesicle origin, cargo composition and the local inflammatory microenvironment. Therefore, miRNA stability in AH should be considered a relative rather than absolute property. These properties make AH-derived EVs an attractive source of biomarkers for the investigation of local cellular and immune processes in intraocular disease (Dismuke et al., 2015; Liu et al., 2020; Aziziyan et al., 2026).

Several studies have investigated the molecular cargo of AH-derived EVs in ocular diseases. A major advance came from Dismuke et al., who characterized vesicles isolated from human AH and reported an abundant population of small EVs, referred to as exosomes in the original study, containing multiple small RNAs and mature miRNAs. This work provided an important methodological precedent for subsequent investigations of AH-derived EV-associated biomarkers (Dismuke et al., 2015).

In neovascular age-related macular degeneration (AMD), Kang et al. compared proteins identified in retinal pigment epithelium-conditioned medium, EV fractions reported as exosomes from conditioned medium, and analogous vesicle fractions isolated from the AH of affected patients using liquid chromatography-tandem mass spectrometry. Subsequent targeted analysis of whole AH from patients and controls identified increased levels of six candidate proteins, including cathepsin D and cytokeratin 8. These findings suggested that disease-associated proteins released by ocular tissues may be transported within EVs and detected in AH (Kang et al., 2014).

In Vogt-Koyanagi-Harada disease (VKH) and Behçet disease (BD), Zhang et al. performed label-free quantitative proteomic analysis of AH-derived small EVs, referred to as exosomes in the original study, from 84 patients. The study identified 65 differentially expressed proteins in VKH disease and 40 in BD, with functional enrichment predominantly involving complement-related pathways. Complement C1q subcomponent subunit B (C1QB) was consistently enriched in the isolated EV fractions from both diseases and emerged as a key candidate biomarker. Integration with previously published single-cell RNA sequencing data further suggested that C1QB-containing EVs are primarily derived from mononuclear macrophages residing in anterior segment tissues (Zhang et al., 2024).

Collectively, these studies support the potential of AH-derived EVs as a source of molecular biomarkers. However, disease-specific characterization of their miRNA cargo remains relatively unexplored, particularly in infectious ocular diseases (**Table 1**). Thus, although AH-derived EVs have demonstrated biomarker potential in non-infectious ocular diseases, their diagnostic or prognostic value in HSV-1 anterior uveitis remains entirely unvalidated.

**Table 1:** Studies investigating extracellular vesicles and microRNAs in ocular diseases relevant to biomarker discovery.

Disease/Study	Biological sample	EV/miRNA target	Methodology	Main findings	Relevance to HSV-1 AU
Healthy eyes – Dismuke et al. (2015)	Aqueous humor	EV-associated small RNAs and miRNAs	Ultracentrifugation; small RNA analysis	Characterized AH-derived small EVs and identified multiple mature miRNAs.	Established a methodological precedent for AH EV-associated miRNA profiling.
Neovascular AMD – Kang et al. (2014)	Aqueous humor	EV-associated proteins	LC-MS/MS proteomics	Identified candidate disease-associated proteins in vesicle fractions isolated from AH.	Supports the ability of AH EVs to carry disease-associated molecular cargo.
VKH disease and Behçet disease – Zhang et al. (2024)	Aqueous humor	EV-associated proteome	Label-free quantitative proteomics	Identified disease-associated EV protein signatures, including enrichment of complement-related pathways and C1QB.	Supports the concept that AH EVs can reflect local intraocular immune processes.
Behçet disease, VKH disease and sarcoidosis – Asakage et al. (2020)	Serum	Circulating miRNAs	miRNA profiling; machine-learning analysis	Identified disease-associated circulating miRNA signatures capable of discriminating among inflammatory uveitic entities.	Demonstrates the biomarker potential of systemic miRNAs while highlighting the need for compartment-specific profiling.
Immune recovery uveitis – Duraikkannu et al. (2023)	PBMCs	Cellular miRNAs	miRNA expression profiling	Reduced miR-192-5p and miR-543 were associated with subsequent development of immune recovery uveitis.	Supports the potential predictive value of miRNA signatures in ocular inflammation.
Behçet uveitis – Jiang et al. (2023)	Plasma-derived EVs	EV-associated miR-19b-3p	Small RNA sequencing; functional assays	miR-19b-3p promoted Th17 differentiation and impaired Treg differentiation through CD46 regulation.	Demonstrates that EV-associated miRNAs may participate directly in immune regulation.
Acute anterior uveitis – Yang et al. (2017)	Peripheral blood	miRNA copy-number variants	CNV analysis; functional validation	Several miRNA loci were associated with disease susceptibility and inflammatory cytokine production.	Supports the involvement of miRNA regulatory pathways in anterior segment inflammation.
JIA-associated uveitis – Sharon et al. (2025)	PBMCs	Cellular miRNAs	Small RNA sequencing	miR-4485-3p was increased in patients with ocular involvement.	Suggests that systemic miRNA profiles may identify ocular involvement but may not directly reflect intraocular events.
Intraocular tuberculosis – Chadalawada et al. (2022)	Aqueous humor	Total AH miRNAs	Ultra-low-input small RNA sequencing; RT-qPCR	Identified 56 differentially expressed miRNAs despite limited AH volume, with selected candidates subsequently validated by RT-qPCR.	Provides proof of principle for low-volume AH miRNA profiling in infectious uveitis, although EV-associated miRNAs were not specifically analyzed.

## MicroRNA networks in HSV-1 infection

Among the molecular cargoes transported by EVs, miRNAs are of particular interest because of their ability to regulate gene expression in recipient cells (Pockar et al., 2019). miRNA cargo of EVs is not simply a passive reflection of the intracellular miRNA repertoire. Specific miRNAs can be selectively incorporated into small EVs through regulated sorting mechanisms, generating molecular profiles that differ from both total cellular RNA and unfractionated biofluids. This distinction is particularly relevant when interpreting AH-derived molecular signatures, as EV-associated miRNAs represent a selectively packaged subset rather than the total extracellular miRNA population (Villarroya-Beltri et al., 2013; Santangelo et al., 2016; Shurtleff et al., 2016; Garcia-Martin et al., 2022).

During HSV-1 infection, both host- and virus-derived miRNAs contribute to the control of viral replication, latency and inflammatory responses (Duan et al., 2023).

During productive infection, HSV-1 alters host miRNA biogenesis while also expressing its own viral miRNAs. Pan et al. demonstrated that lytic infection impairs the nuclear export of cellular precursor miRNAs (pre-miRNAs), leading to their nuclear retention and reduced maturation, whereas viral pre-miRNAs appear to largely escape this restriction (Pan et al., 2019).

HSV-1-encoded miRNAs are processed through the host miRNA machinery. The HSV-1 miRNA repertoire extends beyond miRNAs arising directly from the LAT region and shows substantial variation according to the stage of infection. Among these, miR-H1 is located upstream of the LAT transcriptional start site and is abundantly expressed during productive infection, whereas miR-H3 and miR-H4 are encoded within the LAT region and are among the more abundant viral miRNAs detected during latency (Duan et al., 2023). Experimental studies have also implicated miR-H3 and miR-H4 in the regulation of viral and host pathways, including modulation of ICP34.5 and TGF-β/SMAD signaling, although their precise functions appear to depend on the experimental model (Duan et al., 2023). Importantly, ocular HSV-1 infection may involve a broader repertoire of viral miRNAs: recent experimental work identified miR-H1, miR-H3, miR-H4, miR-H6 and several other HSV-1-encoded miRNAs in infected corneal tissue during active disease (Patil et al., 2025). These observations support the possibility that viral miRNA profiles vary between latent and productive infection and may provide molecular information beyond that captured by individual LAT-associated miRNAs. Umbach et al. identified the latency-associated transcript (LAT), the predominant viral transcript expressed during latency, as the principal precursor of multiple HSV-1 miRNAs (Umbach et al., 2008). Among the best characterized, miR-H2-3p is transcribed antisense to ICP0 and represses its translation without substantially affecting mRNA abundance. Because ICP0 is involved in lytic gene expression and viral reactivation, miR-H2-3p has been implicated in the regulation of the latency-reactivation balance, although its biological effects vary across experimental models. miR-H6 similarly targets ICP4, an immediate-early transcription factor required for productive viral gene expression, thereby providing an additional level of post-transcriptional control over the lytic program (Umbach et al., 2008).

Beyond regulating immediate-early viral genes, HSV-1 miRNAs also target host cellular pathways that facilitate viral replication. HSV-miR-H27 suppresses the host transcriptional repressor KLHL24, thereby relieving inhibition of viral immediate-early gene expression and promoting viral replication (Umbach et al., 2008). These findings demonstrate that HSV-1-encoded miRNAs regulate both viral and host transcripts, enabling the virus to fine-tune the transition between latency and productive infection.

Host-derived miRNAs also influence the outcome of HSV-1 infection. The neuron-specific miR-138 directly targets ICP0, suppresses lytic gene expression and promotes latency. The regulation of ICP0 by both cellular miR-138 and viral miR-H2-3p further emphasizes the importance of this immediate-early protein in the latency–reactivation switch (Pan et al., 2019).

Distinct HSV-1-encoded miRNAs show different associations with latent and productive infection. miR-H2, miR-H3 and miR-H4 are associated with the LAT region and have been linked predominantly to latency-related regulation, whereas miR-H6, although encoded outside LAT, also contributes to latency by targeting ICP4. In contrast, miR-H27 has been associated with active viral replication and reactivation (Duan et al., 2023; Scheiber et al., 2024).

Host-derived miRNAs can also modulate inflammatory signaling pathways during HSV-1 infection, thereby influencing the magnitude and composition of the ocular immune response (Mulik et al., 2012; Suryawanshi et al., 2015; Rajasagi et al., 2017). Among them, miR-155 is markedly upregulated during HSV-1 infection and plays a central role in the development of herpetic stromal keratitis. In experimental models, both genetic deletion and pharmacological inhibition of miR-155 reduce corneal inflammation, Th1/Th17 responses, neovascularization, and disease severity, identifying miR-155 as a promising therapeutic target (Suryawanshi et al., 2015). In contrast, miR-132 has been implicated primarily in HSV-induced corneal neovascularization. Ocular HSV-1 infection induces marked upregulation of miR-132 through VEGF-A- and IL-17-dependent signaling. In experimental models, pharmacological inhibition of miR-132 with antagomir-132 significantly reduces corneal neovascularization and stromal keratitis severity, supporting its role as a regulator of angiogenesis and a potential therapeutic target in HSV-induced ocular disease (Mulik et al., 2012).

The resolution of HSV-induced ocular inflammation is also influenced by specialized pro-resolving mediators (SPMs). In a murine model of herpetic stromal keratitis, treatment with aspirin-triggered resolvin D1 (AT-RvD1), an epimer of resolvin D1 derived from docosahexaenoic acid, significantly reduced corneal neovascularization and disease severity. These effects were associated with decreased infiltration of neutrophils and Th1/Th17 cells, reduced expression of pro-inflammatory cytokines and angiogenic mediators, including IL-1β, IL-6, IL-12, VEGF-A and MMP-9, and downregulation of miR-155, miR-132 and miR-223, all of which contribute to HSV-induced corneal immunopathology. Collectively, these findings suggest that modulation of SPM-dependent miRNA networks may represent a promising therapeutic strategy for limiting HSV-induced ocular inflammation (Rajasagi et al., 2017).

HSV-1 infection may also influence the biogenesis and molecular composition of extracellular vesicles. EV formation involves multiple pathways, including endosomal sorting mechanisms and ESCRT-dependent and ESCRT-independent processes (van Niel et al., 2018). More broadly, virus-infected cells can release EVs containing both host- and virus-derived molecular cargo, indicating that viral infection can alter the composition of extracellular vesicles (Nolte-'t Hoen et al., 2016). Consequently, an HSV-1-associated EV miRNA signature could reflect not only changes in intracellular miRNA expression but also selective incorporation of host- or virus-derived RNAs into released vesicles. Whether HSV-1 specifically modifies EV biogenesis or miRNA cargo sorting within the anterior chamber remains unknown.

The concept of EV-associated miRNAs as markers of host–virus interactions is supported by observations in other viral infections. In EBV infection, EVs released from infected cells can contain viral miRNAs and other virus-derived molecules capable of modifying recipient-cell signaling (Cone et al., 2019). HCMV-encoded miRNAs have also been detected in the circulation, including within extracellular vesicular fractions, and have been investigated as potential biomarkers of viral and disease-associated processes (Wang et al., 2019). Similarly, EVs have been implicated in HIV persistence, immune regulation and host–virus communication through the transfer of host- and virus-derived molecular cargo (Salzano et al., 2026). Together, these observations provide a broader virological precedent for investigating EV-associated miRNAs in HSV-1 infection, although findings from systemic infections cannot be directly extrapolated to the aqueous humor compartment.

However, current evidence derives mainly from neuronal tissues, corneal infection models and experimental systems. Whether comparable alterations in EV biogenesis and host- or virus-derived EV-associated miRNA profiles occur within the AH during intraocular HSV-1 infection remains unknown.

## Circulating and EV-associated miRNA biomarkers in uveitis

Most evidence on miRNA biomarkers in uveitis has been generated from peripheral blood rather than intraocular samples. Several studies have investigated circulating miRNAs as minimally invasive biomarkers, providing important insights into disease-specific immune pathways.

One of the earliest analyses of circulating miRNAs in non-infectious uveitis was conducted by Asakage et al., who profiled serum miRNA expression in patients with BD, sarcoidosis and VKH. Distinct disease-specific signatures were identified, with miR-4708-3p, miR-4323 and let-7g-3p showing the highest diagnostic performance for BD, sarcoidosis and VKH disease, respectively. Pathway enrichment analysis associated these miRNAs with MAPK signaling, cytokine regulation and inflammatory pathways, while machine-learning algorithms further improved disease classification, highlighting the potential of circulating miRNA panels for differential diagnosis (Asakage et al., 2020).

Additional evidence supporting the biomarker potential of circulating miRNAs comes from immune recovery uveitis (IRU) in HIV-infected patients. Reduced expression of miR-192-5p and miR-543 in peripheral blood mononuclear cells (PBMCs) distinguished patients with IRU from HIV-infected individuals without ocular involvement. Bioinformatic analyses indicated that both miRNAs regulate multiple inflammatory signaling pathways, supporting their potential as biomarkers of immune-mediated ocular inflammation (Duraikkannu et al., 2023).

Beyond cell-free circulating miRNAs, plasma-derived EV-associated miRNAs have provided important insights into the immunopathogenesis of ocular inflammation. In patients with active Behçet’s uveitis, miRNA sequencing identified marked dysregulation of plasma EV-associated miRNAs compared with healthy controls. Among these, miR-19b-3p was significantly upregulated and was shown to promote Th17 differentiation while suppressing regulatory T-cell differentiation through downregulation of CD46. Conversely, inhibition of miR-19b-3p restored the Treg/Th17 balance, supporting a functional role for EV-associated miRNAs in the immunopathogenesis of Behçet’s uveitis (Jiang et al., 2023).

Further evidence implicating miRNA pathways in uveitis derives from genetic studies. In patients with acute anterior uveitis (AAU), copy number variations involving miR-143, miR-146a, miR-9-3, miR-205, miR-301a, and miR-23a were significantly associated with disease susceptibility. Among these, reduced miR-9–3 copy number correlated with lower miRNA expression, and functional studies demonstrated that miR-9–3 regulates IL-1β and IL-6 production in human retinal pigment epithelial cells. These findings provide a mechanistic link between genetic variation in miRNA loci and ocular inflammatory responses (Yang et al., 2017).

Evidence from juvenile idiopathic arthritis-associated uveitis (JIA-U) further supports the potential of systemic miRNA profiling. Small RNA sequencing of peripheral blood mononuclear cells identified significantly increased expression of miR-4485-3p in patients with JIA-U compared with those with juvenile idiopathic arthritis without ocular involvement. Although miR-4485-3p was not associated with disease activity, its differential expression suggests potential utility as a biomarker for identifying patients at increased risk of uveitis, pending validation in larger cohorts (Sharon et al., 2025).

Collectively, these studies establish circulating and EV-associated miRNAs as promising biomarkers of immune-mediated uveitis, reflecting disease susceptibility, immune activation and inflammatory pathways. However, their applicability to HSV-1 anterior uveitis remains limited. Unlike BD, VKH disease, sarcoidosis or JIA-U, HSV-1 anterior uveitis results from a localized interaction between viral replication and host immunity within the immune-privileged anterior chamber of the eye. Consequently, peripheral miRNA signatures are unlikely to accurately capture the compartmentalized molecular events underlying viral persistence, recurrent inflammation and tissue injury.

## Intraocular miRNAs in infectious uveitis

While circulating miRNAs have been widely explored in uveitis associated with systemic diseases, evidence on AH miRNAs is limited. To date, intraocular tuberculosis seems to be among the few infectious uveitides to have undergone comprehensive AH miRNA profiling, providing proof of concept for localized molecular characterization in infectious uveitis.

Using ultra-low-input small RNA sequencing, Chadalawada et al. profiled miRNAs from AH samples obtained from patients with intraocular tuberculosis despite the limited sample volume. The study identified 56 differentially expressed miRNAs, demonstrating both the technical feasibility and analytical sensitivity of next-generation sequencing for comprehensive miRNA profiling in AH. Subsequent quantitative RT-PCR validation confirmed differential expression of several candidate biomarkers, including miR-423-5p, miR-328-3p, miR-21-5p and miR-16-5p. Functional enrichment analyses further linked these miRNAs to biological pathways central to tuberculosis pathogenesis, including PI3K-Akt signaling, autophagy and MAPK signaling, thereby connecting aqueous humor molecular signatures with intracellular host defense mechanisms.

This work indicates that AH miRNA profiling may provide information beyond conventional biomarker discovery. By capturing both pathogen-induced and host-derived molecular responses directly within the immune-privileged intraocular environment, it provides mechanistic insights into disease pathogenesis that cannot be obtained through conventional microbiological testing or peripheral blood analyses. This study provides proof of principle that comprehensive miRNA profiling is technically achievable in low-volume AH samples from infectious uveitis (Chadalawada et al., 2022). However, because the analysis was performed on total AH miRNAs rather than EV-associated miRNAs, these findings do not establish the feasibility of AH EV-associated miRNA profiling in HSV-1 anterior uveitis. Instead, they provide an important methodological precedent supporting further investigation of localized miRNA signatures in ocular infection.

## Methodological challenges in AH-derived EV-associated miRNA profiling

Although advances in EV research and low-input RNA analysis provide a methodological basis for investigating AH-derived EV-associated miRNAs, several technical challenges must be addressed before this approach can be reliably evaluated and translated into clinical practice (Mateescu et al., 2017; Théry et al., 2018).

One of the principal limitations is the extremely small volume of AH that can be safely obtained during anterior chamber paracentesis, typically ranging from 100 to 200 μL. This may restrict both EV yield and RNA recovery, requiring highly sensitive isolation and sequencing protocols specifically optimized for low-input samples (Mateescu et al., 2017; Van Deun et al., 2017).

Pre-analytical, clinical and pharmacological variables represent important potential sources of variability in AH EV-associated miRNA studies. The timing of sample collection and level of inflammatory activity should be standardized, as the cellular environment and pathological state can influence EV release and molecular cargo (Mateescu et al., 2017; Van Deun et al., 2017; Théry et al., 2018; Jeppesen et al., 2019). Ongoing or recent treatment should also be systematically recorded, including topical versus systemic antiviral therapy and topical, periocular or systemic corticosteroids, together with treatment duration and timing relative to AH sampling. Although the direct effects of these therapies on AH EV-associated miRNA profiles have not been established, treatment-related modulation of viral replication and intraocular inflammation may confound the molecular signature under investigation.

Additional clinical variables should include first versus recurrent episodes, duration of inflammation, anterior chamber cell and flare, concomitant keratitis, active co-infection, intraocular pressure and corneal endothelial status. Previous or concurrent ocular procedures, particularly glaucoma surgery or keratoplasty, should also be considered because the underlying disease state and surgical indication may themselves alter the local ocular microenvironment. These variables should be prospectively recorded and incorporated into study design and statistical analysis to distinguish HSV-1-associated molecular signals from treatment- or disease-related effects.

A significant challenge will be defining whether identified miRNA signatures are specific to HSV-1 infection or instead reflect intraocular inflammation more broadly. Many miRNAs implicated in HSV-1 infection also participate in general inflammatory, angiogenic and immune-regulatory pathways, and therefore differential expression in AH may represent a response to tissue injury or inflammation rather than a virus-specific molecular signature. Future studies should therefore include appropriate comparator groups, including other infectious and non-infectious causes of anterior uveitis, to determine the specificity of candidate miRNA signatures for HSV-1 infection.

Additional challenges arise from the lack of standardized EV isolation methods. Differential ultracentrifugation, polymer-based precipitation, size-exclusion chromatography, ultrafiltration and immunoaffinity-based approaches differ substantially in terms of recovery, purity and reproducibility. Consequently, the molecular profiles obtained may vary according to the isolation strategy employed, highlighting the need for standardized protocols (Mateescu et al., 2017; Van Deun et al., 2017; Jeppesen et al., 2019).

EV heterogeneity represents an additional challenge for biomarker interpretation. Biological fluids contain mixed populations of extracellular vesicles that differ in size, biogenesis and cellular origin, and currently available isolation methods generally enrich rather than completely purify individual EV subtypes (Mateescu et al., 2017; Van Deun et al., 2017; Théry et al., 2018; Jeppesen et al., 2019). Consequently, preparations enriched for small EVs may also contain other vesicular populations and non-vesicular extracellular components, including protein or ribonucleoprotein complexes. This is particularly relevant in low-volume biofluids such as AH, where extensive purification may further reduce EV and RNA yield. Therefore, differences in detected miRNA profiles may reflect not only disease biology but also EV population heterogeneity and isolation-related bias.

Normalization represents a further challenge in low-input EV-associated miRNA studies. Common approaches include the use of exogenous spike-in RNAs, normalization to EV particle number, total RNA input, global mean miRNA expression, or selected endogenous reference miRNAs (Mateescu et al., 2017; Pinheiro et al., 2024). However, each strategy has important limitations. Spike-in controls can account for technical variability during RNA extraction and reverse transcription but do not correct for biological differences in EV abundance between samples (Mateescu et al., 2017). Normalization to particle number depends on accurate EV quantification and may be affected by co-isolated non-vesicular particles, whereas endogenous miRNAs may themselves vary with disease state or inflammation (Pinheiro et al., 2024). In addition, normalization to total RNA can be problematic in low-volume AH samples because RNA concentrations may approach the analytical detection limit. No universally accepted normalization strategy currently exists for EV-associated miRNA analysis; therefore, the selected approach should be predefined, justified and consistently applied across samples (Mateescu et al., 2017; Théry et al., 2018).

Addressing these methodological issues will be essential to improve reproducibility across studies and to enable meaningful comparisons between patient cohorts. Adoption of standardized workflows in accordance with the Minimal Information for Studies of Extracellular Vesicles (MISEV) recommendations will represent an important step toward the clinical application of AH-derived EV-associated miRNA profiling. These factors should also be considered when comparing studies that use total AH RNA with those specifically analyzing extracellular vesicle-associated miRNAs (Théry et al., 2018).

## Future perspectives: AH-derived EV-associated miRNAs in HSV-1 anterior uveitis

A major unmet need in HSV-1 anterior uveitis is the identification of biomarkers that provide information beyond viral DNA detection. To date, diagnostic confirmation relies primarily on PCR analysis of AH, which establishes the presence of HSV-1 DNA but provides limited insight into the molecular composition of the intraocular environment.

The physiological acellularity of AH provides a strong biological rationale for investigating EVs. Under physiological conditions, EVs constitute one of the principal sources of nucleic acids and proteins within the aqueous compartment, whereas during intraocular inflammation they are released by both resident ocular cells and infiltrating inflammatory cells. Characterization of their miRNA cargo may expand the molecular information obtained from the same AH sample collected for selected diagnostic investigations. An important distinction should be made between total AH miRNAs and EV-associated miRNAs. Total AH miRNA measurements capture a heterogeneous extracellular pool that may include vesicle-associated miRNAs as well as miRNAs associated with proteins or other extracellular carriers. By contrast, EV-associated miRNAs represent a more selectively packaged molecular fraction, as specific miRNAs can be preferentially incorporated into EVs through regulated sorting mechanisms (Villarroya-Beltri et al., 2013; Santangelo et al., 2016; Shurtleff et al., 2016; Garcia-Martin et al., 2022; Verma et al., 2025). EV encapsulation may also provide relative protection from extracellular degradation and may preserve information related to the cellular source and biological state of the releasing cells. However, EV enrichment introduces additional pre-analytical and analytical variability and may further reduce RNA yield in low-volume AH samples. Therefore, EV-associated miRNAs should not currently be considered analytically superior to total AH miRNAs; rather, they represent a biologically distinct compartment whose incremental diagnostic value requires direct comparison with unfractionated AH miRNA profiling.

The successful application of AH miRNA profiling in intraocular tuberculosis has demonstrated that comprehensive molecular characterization is feasible despite the limited sample volumes obtained by anterior chamber paracentesis. Together with the established role of host- and virus-derived miRNAs in HSV-1 infection, these findings provide a strong biological and methodological rationale for investigating AH-derived EV-associated miRNAs in HSV-1 anterior uveitis. However, this rationale remains hypothesis-generating, as the presence, differential expression and clinical utility of EV-associated miRNAs in the AH of individuals with HSV-1 anterior uveitis is yet to be established.

A key objective of future studies should be to determine whether EV-associated miRNA profiles differ according to the biological state of HSV-1 infection. This question would be best addressed through longitudinal designs incorporating AH sampling at clinically relevant time points, including active disease, early treatment and, when available, subsequent quiescent or recurrent episodes. EV-associated miRNA profiles should be analyzed together with HSV-1 PCR results, quantitative viral load where available, anterior chamber inflammatory activity, intraocular pressure, corneal involvement and treatment exposure. Parallel profiling of both virus-derived and host-derived miRNAs could then be used to assess whether specific signatures are associated with active viral replication, reactivation or clinically quiescent disease. Importantly, paired analysis of EV-associated and total AH miRNAs within the same samples would help determine whether EV enrichment provides additional biological or diagnostic information beyond unfractionated AH profiling. Such longitudinal within-patient comparisons would be particularly valuable because they reduce inter-individual biological variability and allow assessment of whether candidate miRNAs track changes in disease activity over time.

## Clinical implications and translational potential of EV-associated miRNAs in HSV-1 anterior uveitis

While the clinical utility of AH-derived EV-associated miRNAs in HSV-1 anterior uveitis has not yet been established, the available biological and methodological evidence suggests that they may have translational potential as candidate biomarkers. However, this potential remains hypothetical until disease-associated miRNA signatures are identified, technically validated and independently evaluated in clinically well-characterized cohorts. If validated, EV-associated miRNA profiling could expand the information obtained from conventional diagnostic testing by providing molecular characterization of the intraocular environment in addition to viral DNA detection. Unlike inflammatory cells, EVs are constitutively present in the physiologically acellular AH, raising the possibility that clinically informative molecular signatures could be obtained even from samples collected in eyes with minimal or absent anterior chamber inflammation.

From a practical perspective, EV-associated miRNA analysis could potentially be performed using residual AH obtained during clinically indicated anterior chamber paracentesis, thereby avoiding additional sampling solely for biomarker analysis. However, whether the remaining sample volume is sufficient for reproducible EV isolation and miRNA profiling after routine diagnostic testing has not yet been established. This will require prospective evaluation using standardized low-input workflows before such an approach can be considered clinically feasible.

Beyond diagnosis, characterization of AH-derived EV-associated miRNAs may contribute to a better understanding of the molecular mechanisms underlying HSV-1 anterior uveitis. Integration of molecular profiling with virological findings, cytokine analysis, multimodal imaging and clinical phenotyping could help identify disease-associated molecular signatures and improve biological characterization of the disease. Whether these signatures ultimately prove useful for diagnosis, prognosis or therapeutic monitoring remains to be established in future clinical studies.

Translation of EV-associated miRNA profiling into clinical practice will require several additional steps beyond biomarker discovery. Analytical validation should first establish assay reproducibility, sensitivity, specificity and robustness across different laboratories and technical platforms (Mateescu et al., 2017; Van Deun et al., 2017; Théry et al., 2018). Pre-analytical procedures, EV isolation methods, RNA extraction protocols and normalization strategies will also need to be standardized to reduce inter-laboratory variability (Mateescu et al., 2017; Van Deun et al., 2017; Théry et al., 2018). Candidate signatures should then undergo independent validation in prospectively recruited and clinically well-characterized cohorts, ideally across multiple centers. In addition, clinically meaningful thresholds and the incremental diagnostic value of EV-associated miRNA profiling over established tests, particularly AH PCR, will need to be defined. Finally, any assay intended for routine clinical use would require appropriate quality-control procedures and compliance with applicable regulatory requirements before implementation.

Future studies should distinguish biomarker discovery from clinical validation. Initial studies should determine whether specific EV-associated miRNA signatures differ between patients with HSV-1 anterior uveitis and appropriately selected comparator groups, followed by independent technical and clinical validation. Comparator cohorts should include VZV-associated anterior uveitis, which provides a biologically relevant comparison with another alphaherpesvirus; CMV-associated anterior uveitis, which allows assessment against a distinct herpesvirus with different patterns of viral replication and host response; and non-infectious anterior uveitis, which is essential to distinguish virus-specific miRNA changes from signatures related to intraocular inflammation more broadly. When ethically and clinically feasible, AH obtained from non-inflammatory ocular conditions may also provide a baseline reference for the physiological EV-associated miRNA profile. Clinical variables including disease activity, previous antiviral and corticosteroid treatment, recurrent versus first episodes, and anterior chamber inflammatory activity should also be incorporated into biomarker analyses (**Figure 1**).

**Figure 1:**
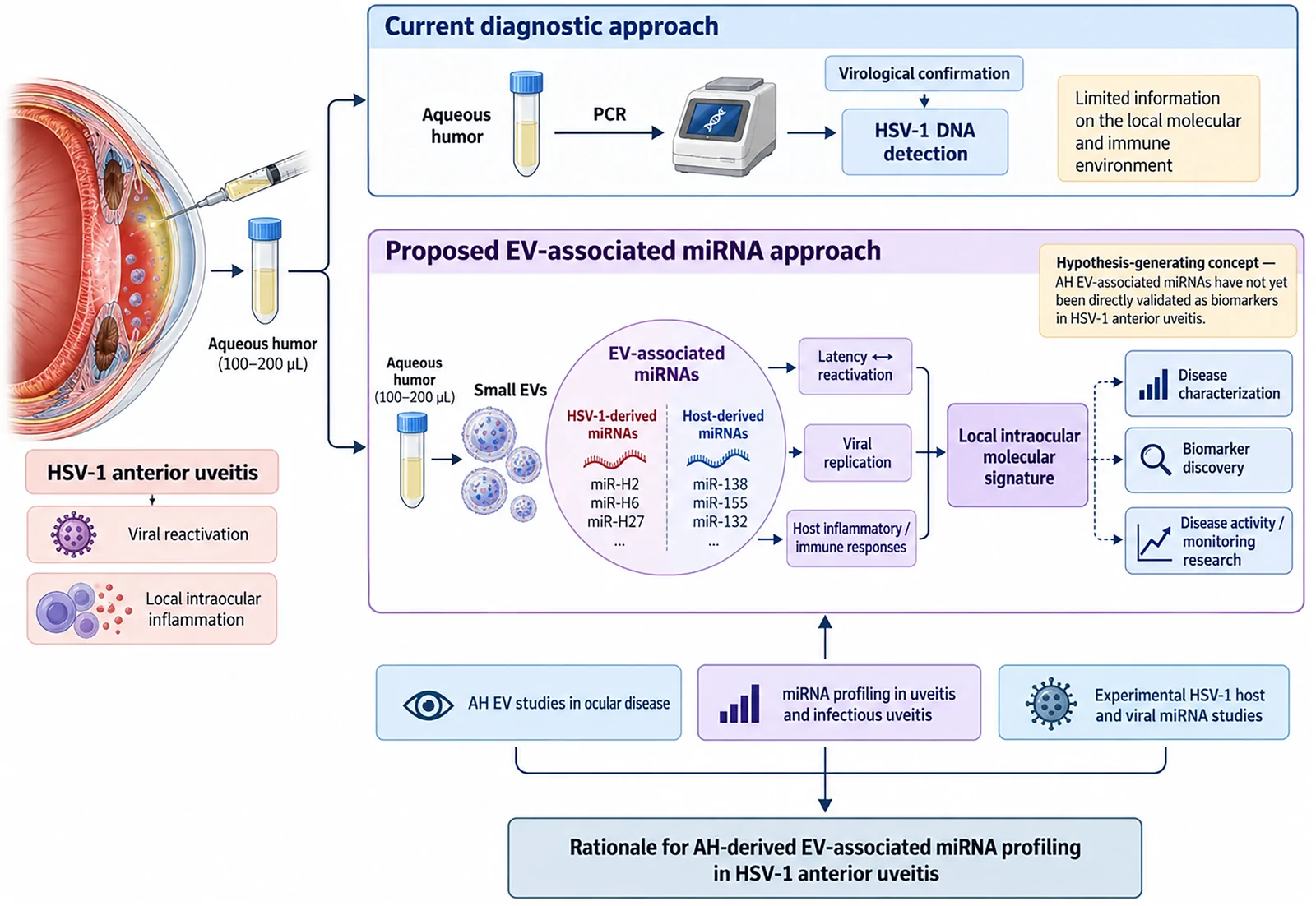
Proposed role of aqueous humor EV-associated miRNA profiling in HSV-1 anterior uveitis. Aqueous humor PCR confirms HSV-1 infection, whereas EV-associated miRNA profiling may provide complementary information on viral activity and host inflammatory responses, supporting biomarker discovery and disease characterization.

## Conclusions

HSV-1 anterior uveitis is characterized by recurrent viral reactivation and compartmentalized intraocular inflammation that are only partially captured by current diagnostic approaches. Throughout this narrative review, we have highlighted the discrepancy between the biological complexity of the disease and the limited information provided by existing clinical tools, which largely rely on PCR testing and structural clinical findings.

Recent advances in EV biology and miRNA research have opened new opportunities for investigating ocular viral diseases at the molecular level. The available evidence suggests that AH-derived EV-associated miRNAs may represent a promising candidate biomarker platform for reflecting local intraocular molecular events. However, this potential remains hypothetical in HSV-1 anterior uveitis, as no study has yet directly characterized AH EV-associated miRNAs in this disease, and their diagnostic, prognostic and therapeutic-monitoring value remains to be determined.
